# Boerhaave Syndrome: A Report of Two Cases and Literature Review

**DOI:** 10.7759/cureus.25241

**Published:** 2022-05-23

**Authors:** Baha Aldeen Bani Fawwaz, Peter Gerges, Gurdeep Singh, Syed Hamaad Rahman, Ahmad Al-dwairy, Arooj Mian, Nihal Khan, Aimen Farooq

**Affiliations:** 1 Internal Medicine, AdventHealth Orlando, Orlando, USA; 2 Medicine, College of Osteopathic Medicine, Kansas City University, Kansas City, USA; 3 Internal Medicine, MedStar Washington Hospital Center, Washington D.C., USA; 4 Internal Medicine, Allama Iqbal Medical College, Lahore, PAK

**Keywords:** pneumothorax (ptx), gastroenterology and endoscopy, endoscopic management, spontaneous esophageal perforation, boerhaave's syndrome

## Abstract

Boerhaave’s syndrome is a rare yet serious condition associated with high mortality and morbidity. Diagnosis of this syndrome is usually done with the aid of imaging and prompt management should be initiated to improve the outcomes. Treatment for this syndrome has been mainly surgical since its discovery by Herman Boerhaave; however, multiple endoscopic approaches have been successfully used recently with the advancement of this field. Here, we describe two cases of Boerhaave’s syndrome that were endoscopically managed along with a brief literature review of the different endoscopic methods used to manage this syndrome.

## Introduction

Esophageal perforation is a rare clinical entity with an estimated incidence of 3.1 per 1,000,000 per year [1]. It is most commonly caused by iatrogenic mechanisms, like endoscopy or surgery-related phenomena, or non-iatrogenic trauma. An extremely rare cause is the effort rupture of the esophagus, aka Boerhaave’s syndrome, which makes up 15% of esophageal perforation cases [2]. This syndrome usually presents with chest pain [3], which can result in a delayed diagnosis as cardiac etiologies are typically pursued initially, particularly in elderly populations. 

Boerhaave’s syndrome presenting with tension pneumothorax is a rare presentation with few documented cases in the literature [4]. We present two unique cases of Boerhaave’s syndrome with distinct presentations and management followed by a discussion on the factors leading to surgical versus endoscopic management of the patients presenting with Boerhaave syndrome and a review of the literature in each case. 

## Case presentation

Case 1

A 63-year-old male patient with a history of histoplasmosis presented to the emergency department (ED) complaining of sudden, right-sided, non-radiating, pleuritic chest pain for an hour, associated with cough, shortness of breath, and palpitations. The patient had been feeling nauseous and reported a few episodes of non-bloody emesis. The patient admitted to actively smoking tobacco and drinking one to two alcoholic drinks per week. 

**Figure 1:**
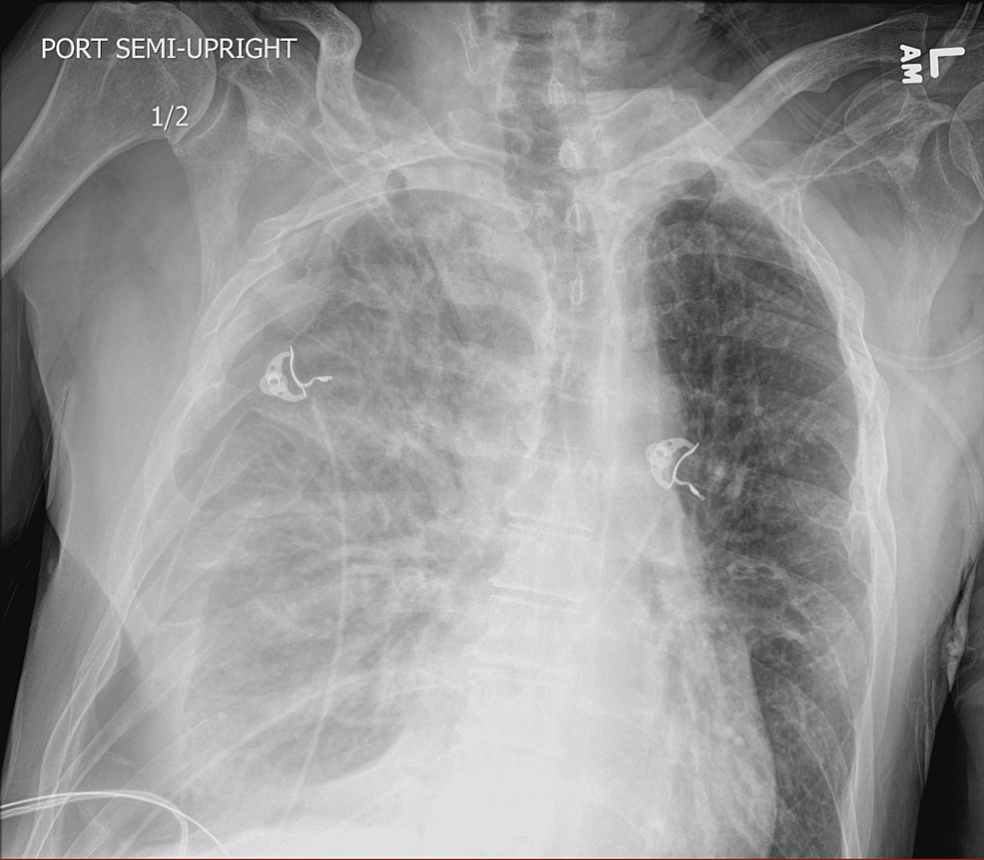
Initial chest x-ray showing right pneumothorax.

On arrival, the patient was afebrile, normotensive (blood pressure (BP) 149/89 mmHg), tachycardiac (heart rate (HR) 108 bpm), tachypneic (respiratory rate (RR) 26 br/min), saturating 93% on ambient air. Stat chest x-ray (CXR) (Figure 1) in ED showed moderate right tension pneumothorax with mass effect on the upper mediastinum and a hazy appearance of the right lung representing atelectasis or pleural effusion. The patient underwent emergent right-sided chest tube placement in the ED with 650mL of dark brown fluid drained. Fluid was exudative per Light’s criteria, bacterial cultures were negative but fungal cultures were positive for *Candida albicans*. The patient was started on broad-spectrum antibiotics and antifungals, and admitted for further management. 

**Figure 2:**
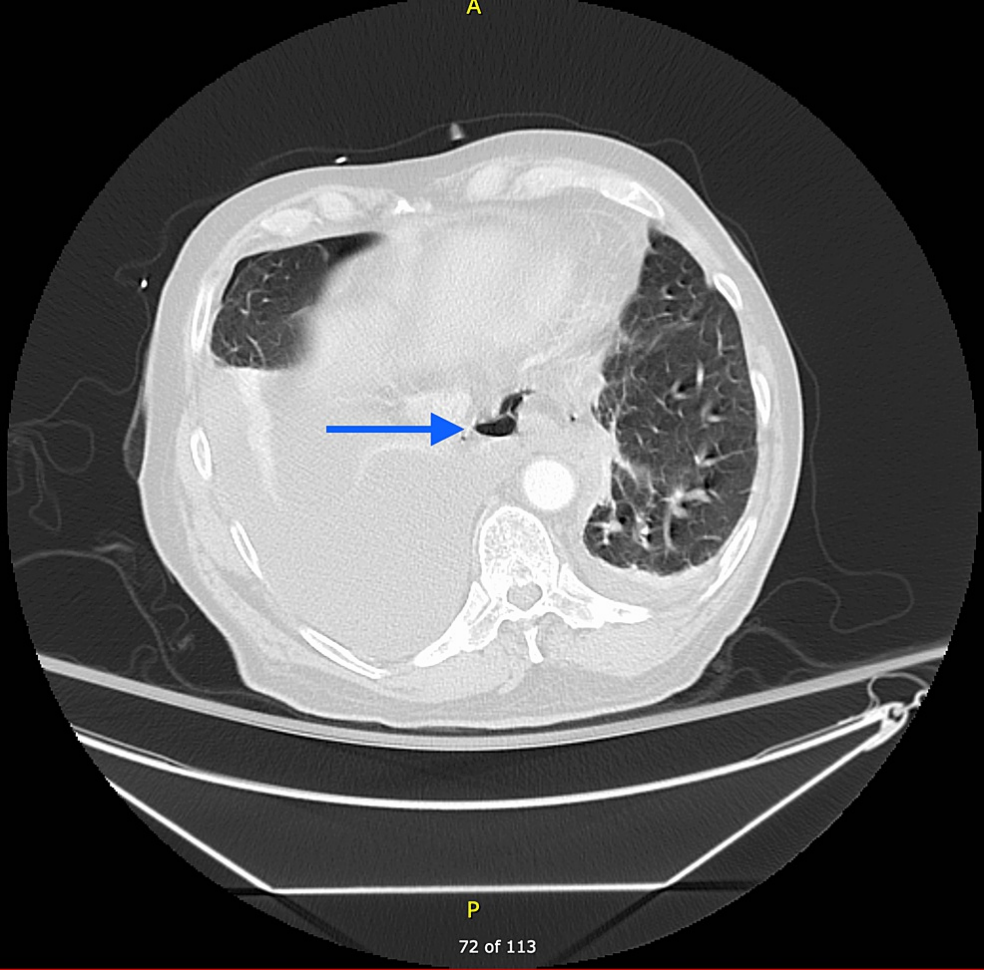
CT chest with contrast showing esophageal thickening with adjacent pneuma-mediastinum.

Labs showed leukocytosis, mild electrolyte derangements, and lactic acidosis. Computed tomography (CT) chest with contrast (Figure 2) showed esophageal thickening with pneumomediastinum adjacent to the distal third of the esophagus concerning for esophageal perforation. Shortly thereafter, the patient developed hypoxic respiratory failure and was transferred to the intensive care unit for mechanical ventilation. After initial resuscitation, the patient underwent esophagogastroduodenoscopy (EGD), as he was a poor surgical candidate, which confirmed the large esophageal perforation 3 cm above the gastroesophageal junction, which was stented with a 23 x 150 mm fully covered self-expanding metal stent (SEMS) under fluoroscopic guidance. An esophagogram performed four days after stenting was negative for leakage. However, the patient had a prolonged course in the hospital secondary to respiratory failure requiring tracheostomy, a repeat EGD was performed three months after for stent removal. Radiocontrast was injected under fluoroscopy, which showed a small transmural defect with a contained pocket of contrast extravasation. It was decided to proceed with the placement of a 23 x 120 mm SEMS under fluoroscopic guidance. The patient was discharged to a long-term acute care facility. 

Case 2

A 56-year-old female with a past medical history of chronic obstructive pulmonary disease (COPD), alcoholic steatohepatitis, chronic alcohol use disorder, and chronic opioid dependence presented with chest pain and shortness of breath. She was tachypneic, tachycardiac, and hypotensive on arrival. EKG revealed inverted T waves in the anterior leads. 

**Figure 3:**
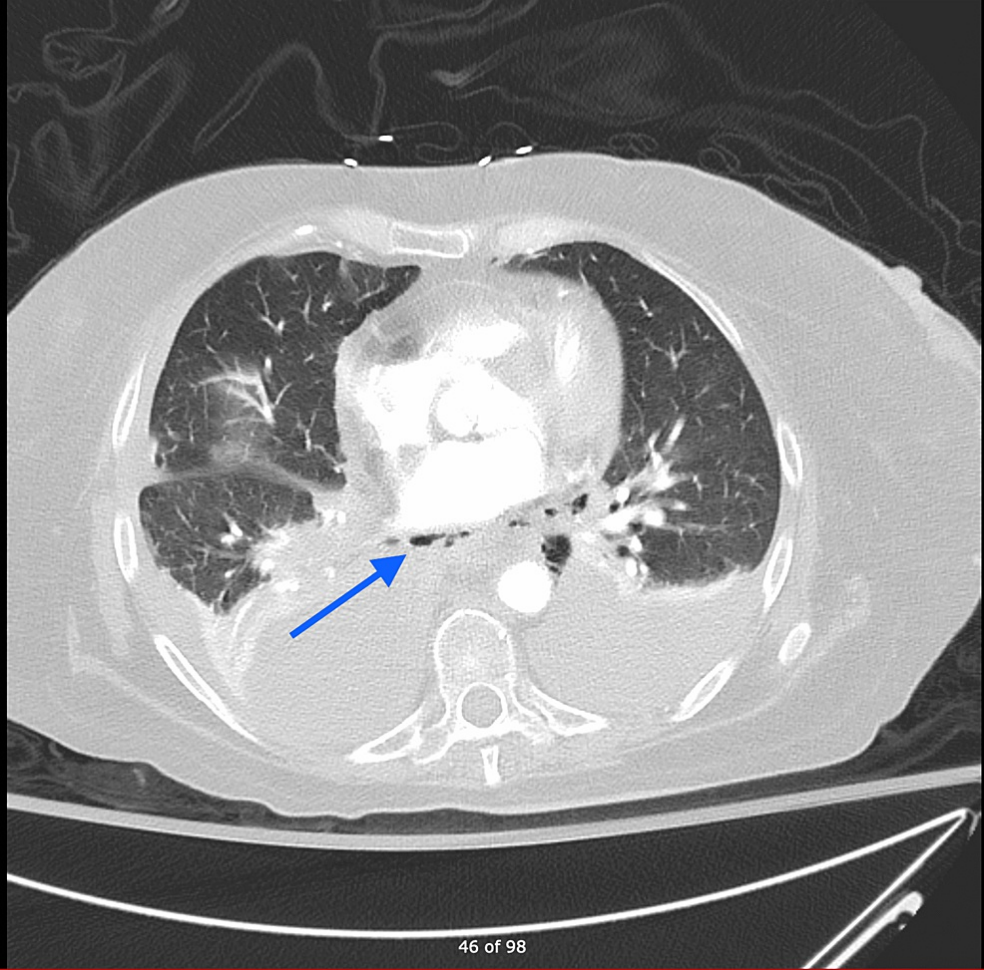
CTA chest showing pneumo-mediastinum. CTA: computed tomography angiography

CXR showed bibasilar airspace opacities. Labs were significant for elevated troponin 0.1 ng/ml (0-0.04 ng/ml) and significantly elevated B-type natriuretic peptide (BNP). After stabilization in ED, the patient underwent cardiac catheterization, which revealed patent coronary arteries. She was transferred to ICU for further management. CT angiography chest (Figure 3) found a massive tear in the distal esophagus. The patient underwent an emergency left thoracotomy, repair of gastroesophageal junction perforation, Belsey fundoplication, T-tube placement, and repair of mid-esophageal perforation with a pericardial patch and required a gastrojejunostomy tube. CT chest showed a loculated right pleural effusion. The patient subsequently went for right video-assisted thoracoscopic surgery with mini-thoracotomy decortication and wash out of the right hemithorax. The postoperative course was complicated by septic shock requiring vasopressors, antibacterial, and antifungal medications. 

**Figure 4:**
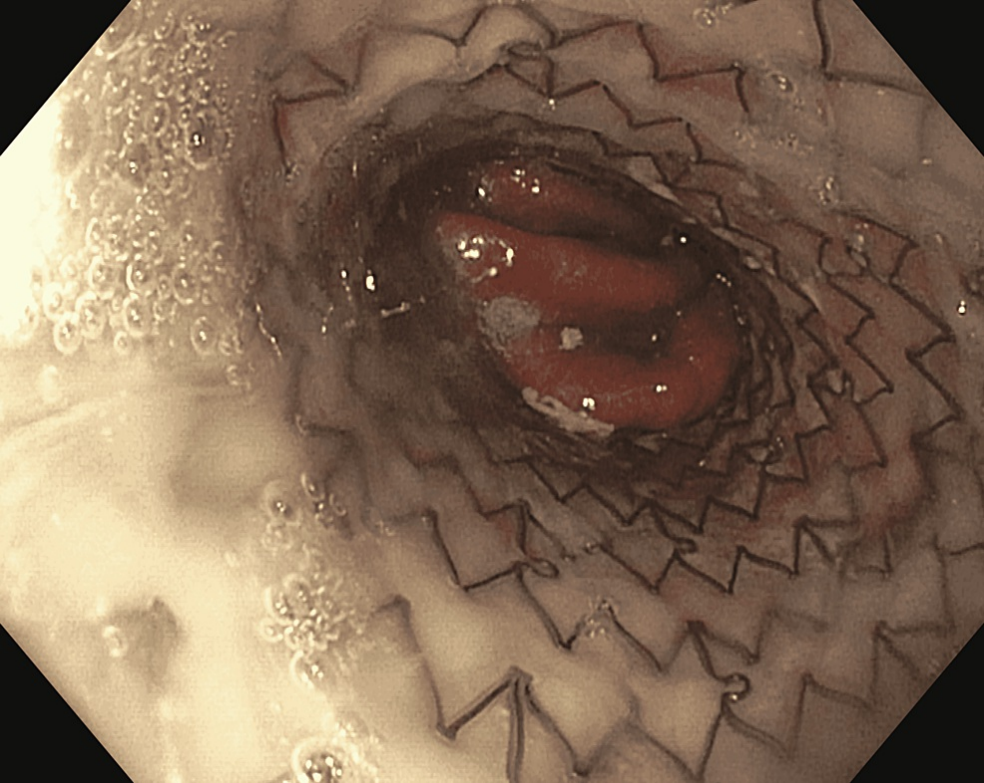
EGD: esophageal stent in place. EGD: esophagogastroduodenoscopy

The patient was deemed not a surgical candidate at this point. A decision was made to proceed with endoscopic evaluation and management of the esophageal perforation. Contrast extravasation was noted during EGD in the distal esophagus. The perforation was managed with a 23 mm x 150 mm EndoMAXX (Merit Medical Systems, Inc., South Jordan, Utah, United States) fully covered self-expanding metal stent (Figure 4) under fluoroscopic guidance. Closure of the defect was confirmed with oral water-soluble contrast at the end of the procedure. The patient had prolonged, complicated hospitalization secondary to empyema of the right chest and a gastrocutaneous fistula eventually requiring surgical intervention. He eventually required a tracheostomy and was discharged to a long-term acute care facility. 

## Discussion

Boerhaave’s syndrome usually occurs due to a sudden increase in the intra-esophageal pressure associated with the negative intrathoracic pressure, causing a traction force that leads to this perforation. This happens in the setting of forceful vomiting and/or retching [5]. Rupture commonly involves the posterolateral part of the distal intrathoracic esophagus [6]. Nonetheless, it can also occur in other parts of the esophagus such as the intra-abdominal or cervical esophagus. This rupture leads to the leakage of the gastric and esophageal components into the mediastinum causing chemical mediastinitis, bacterial infection, necrosis, and death. The mortality rate for untreated Boerhaave’s syndrome can reach 90% [7]. Although the exact morbidity of this syndrome is unknown, it is considered high [8]. 

Management of this syndrome starts with supportive measures including resuscitation, administering broad-spectrum antibiotics and proton pump inhibitors, surgical consultation, as well as possible ICU admission. Subsequent management varies depending on whether the perforation is contained or not. Contained perforations are usually managed medically by avoidance of oral intake, parenteral nutritional support, drainage of fluid collections, and continuing antibiotics. Patients with uncontained perforations or those who fail medical therapy can be managed surgically or endoscopically. 

In our first case description, the patient presented with tension hydropneumothorax, which is an extremely rare presentation [4]. Furthermore, the patient was successfully managed with endoscopic stenting. Traditionally, endoscopic therapy has been reserved for poor surgical candidates. Thus far, only one study was found that compares outcomes of surgically versus endoscopically managed Boerhaave’s syndrome. This study was in 2013, was performed in Europe, and included only 38 patients. It concluded that endoscopic management has no advantage over surgical treatment in terms of morbidity and ICU stay [9]. However, the advancement in the endoscopy field and expertise in recent years, small sample size, and scarcity of studies on this topic might refute that. 

Different endoscopic methods have been used to manage esophageal perforations, including esophageal stents, endoscopic suturing, over-the-scope clips (OTSCs), and through-the-scope clips (TTSCs). The choice of endoscopic intervention depends on the size, location, extent as well as margins of the defect [10]. 

Endoscopic clipping, using OTSCs or TTSCs, is usually preserved for defects that are no greater than 20 mm. The success rate of OTSCs was found to be 100% for esophageal perforations in one multicentric retrospective study, which included 188 patients with GI defects that were managed by OTSCs, 10 of which were esophageal perforations [11]. Esophageal stenting has up to 91.4% technical success rate [12]. Due to the risk of stent migration, careful consideration should be given to the location of the defect. Stent clinical failure has been observed in defects located at upper and lower esophageal sphincters, as well as defects larger than 60 mm in size [13]. Endoscopic suturing also has a comparable success rate of 95.7% in one retrospective study; however, this was for different GI defects and not just esophageal perforations [14]. 

## Conclusions

Overall, the endoscopic management of GI defects in general and esophageal perforation in specific is a promising field that needs further studying and more evidence to structure it and include it in future guidelines. Increasing awareness among physicians about those successful non-surgical options is also needed to improve outcomes of this syndrome.
